# Post-intubation iatrogenic tracheobronchial injuries: The state of art

**DOI:** 10.3389/fsurg.2023.1125997

**Published:** 2023-02-13

**Authors:** Eliseo Passera, Riccardo Orlandi, Matteo Calderoni, Enrico Mario Cassina, Ugo Cioffi, Angelo Guttadauro, Lidia Libretti, Emanuele Pirondini, Arianna Rimessi, Antonio Tuoro, Federico Raveglia

**Affiliations:** ^1^ Department of Thoracic Surgery, San Gerardo Hospital, ASST Monza, Monza, Italy; ^2^ Department of Thoracic Surgery, University of Milan, Milan, Italy; ^3^ Department of Surgery, University of Milan, Milan, Italy; ^4^ Department of Medicine and Surgery, University of Milan-Bicocca, Monza, Italy

**Keywords:** iatrogenic tracheal injury, tracheal surgery, thoracic surgery, endoscopy, tracheobronchial laceration

## Abstract

Iatrogenic tracheobronchial injury (ITI) is an infrequent but potentially life-threatening disease, with significant morbidity and mortality rates. Its incidence is presumably underestimated since several cases are underrecognized and underreported. Causes of ITI include endotracheal intubation (EI) or percutaneous tracheostomy (PT). Most frequent clinical manifestations are subcutaneous emphysema, pneumomediastinum and unilateral or bilateral pneumothorax, even if occasionally ITI can occur without significant symptoms. Diagnosis mainly relies on clinical suspicion and CT scan, although flexible bronchoscopy remains the gold standard, allowing to identify location and size of the injury. EI and PT related ITIs more commonly consist of longitudinal tear involving the pars membranacea. Based on the depth of tracheal wall injury, Cardillo and colleagues proposed a morphologic classification of ITIs, attempting to standardize their management. Nevertheless, in literature there are no unambiguous guidelines on the best therapeutic modality: management and its timing remain controversial. Historically, surgical repair was considered the gold standard, mainly in high-grade lesions (IIIa-IIIb), carrying high morbi-mortality rates, but currently the development of promising endoscopic techniques through rigid bronchoscopy and stenting could allow for bridge treatment, delaying surgical approach after improving general conditions of the patient, or even for definitive repair, ensuring lower morbi-mortality rates especially in high-risk surgical candidates. Our perspective review will cover all the above issues, aiming at providing an updated and clear diagnostic-therapeutic pathway protocol, which could be applied in case of unexpected ITI.

## Introduction

Iatrogenic tracheobronchial injury (ITI) can be defined as any lesion occurring in the airway due to invasive medical or surgical procedure. Main causes are orotracheal intubation and tracheostomy, defining the post-intubation ITIs, and this review will focus on them (1, 2). Other causes include thoracic and neck surgeries but discussing them is beyond the aims of the present review. Globally, post-intubation ITI is considered rare (2), thanks to advancements in medical devices and development of innovative less-invasive procedures; nonetheless its consequences could be awful (3). The recent SARS-CoV-2 pandemic has well raised the issue since the high rate of emergency intubation and close radiological imaging monitoring have brought out several ITI cases (3–5). However, experiences in this field are limited just by the rarity of this condition, and literature is still lacking updated definitive indications on its identification and management. Therefore, the following perspective review aims at discussing the main aspect of post-intubation ITIs, eventually proposing an updated diagnostic-therapeutic algorithm, appliable in case of unexpected ITI.

## Epidemiology

The real incidence of ITIs is unknown, but it is estimated to be 0.005% for all endotracheal (ET) intubation, up to 0.5% for double-lumen tube procedures, and 1% for tracheostomy (6, 7). These rates are likely underestimated, due to several cases are underrecognized or even underreported. Surely, emergency procedures increase the risk for accidental injury; in such settings, the incidence is reported to be up to 15% (2, 8).

## Risk factors

Predisposing factors can be divided into patient-related and procedure-related. The formers are largely unmodifiable and consist of advanced age, female gender, obesity, chronic use of inhaled or systemic steroids, local inflammation and all those conditions leading to tissue malacia (3, 7, 9–11); furthermore, anatomic variations or alterations, such as tracheal diverticula or neoplasms, neck or mediastinal masses dislocating the trachea, marked cervical lordosis or scoliosis, fall into this category (7). The latters include multiple attempts or limited experience in intubation, misuse of a stylet or rigid-guide, as well as incorrect choice of tube or cannula size, double-lumen tube, mishandling of cuff pressure or leverage of the tube (2). Emergency procedures can enhance each of the described risk factors, explaining the higher prevalence of ITIs in emergency settings (12).

## Pathophysiology

Usually, post-intubation ITIs are caused by friction of the endotracheal tube against the pars membranacea of the tracheal wall, at the midline along the posterior membrane or at the cartilaginous-membrane junction, whereas cartilaginous rings and ligaments offer relative protection from injury to the anterior wall (13). Typically, the injury consists of longitudinal tear at the tracheal middle third, which may spread to the lower third or even to the main bronchi: the length is highly variable (14). The depth of the laceration is similarly variable, but it is critical to be assessed. Indeed, based on depth of the lesion, in 2010 Cardillo and colleagues (8) proposed a morphological classification for patients-risk-stratification, aiming at standardizing treatments. According to that classification, post-intubation ITIs were categorized as follows: I, partial-thickness lesion (limited to mucose or submucose) without mediastinal or subcutaneous emphysema; II, full-thickness lesion with mediastinal or subcutaneous emphysema, but without esophageal or mediastinal soft-tissue involvement; IIIA, full-thickness lesion with esophageal or mediastinal soft-tissue herniation, but without esophageal injury or mediastinitis; IIIB, full-thickness lesion with esophageal injury or mediastinitis. Recently this classification has been revised, adding level IV lesions, characterized by extensive loss of substance or fracture of tracheal rings (8, 15, 16).

## Clinical features

Usual clinical presentation consists of facial and upper-trunk subcutaneous emphysema together with cough, occurring within a variable interval of time from ET intubation, generally up to 3 days (14, 17). Dyspnoea can variably occur, from breathing discomfort up to real acute respiratory failure, depending on severity of the lesion and association of unilateral or bilateral pneumothorax (2). ITI may also have asymptomatic course, especially in case of partial-thickness lacerations (14, 17, 18). In mechanical ventilated patients, ITIs can have either subtle development (17), with delayed occurrence in case the cuff overcomes or covers the lesion as well as with ventilatory leaks needing for over-cuffing the tube, or catastrophic presentation (2, 14), with rapid-onset massive pneumomediastinum, tension pneumothorax and difficult ventilation, mainly depending on extent of the tear. Based on mediastinal involvement degree and extent of pneumomediastinum, pneumopericardium, angina or even hypovolemic or cardiogenic shock may occur (4). Haemoptysis or pneumoperitoneum are seldom reported in literature (2, 18, 19).

## Diagnosis

Nowadays, several ITIs are likely misdiagnosed, leading to delayed workup and late treatment, with detrimental effects on patients' outcome (2). To overcome this issue, it is recommended keeping high suspicion in case of suggestive symptoms in patients under mechanical ventilation or with medical history of recent intubation. To define and characterize the suspected lesion, radiologic imaging and endoscopic visualization are two complementary pillars of the diagnostic workup (20). Imaging can be obtained either through chest x-ray or CT-scan. The former allows to promptly rule out pneumothorax, large pneumomediastinum, pneumoperitoneum or subcutaneous emphysema, and it can be very useful in emergency settings to shrink differential diagnosis (21). The latter allows to detect the same findings as x-ray does, with greater sensitivity and accuracy (20–22).

Furthermore, contrast-enhanced CT scan may directly reveal tracheal laceration, approximately defining its site and extent, assessing alterations or deformities of the tracheal wall and cartilaginous rings, as well as identifying collateral damages to mediastinal organs or mediastinitis (12, 23). Typically, a tracheal tear may be highlighted as follows: discontinuity in the tracheal wall, localized pneumomediastinum, overdistension or herniation of the cuff, or tube displacement (24); in case of laceration expanding towards a main bronchus, the fallen lung sign could be noted (25). Another crucial role of the CT scan is to provide a non-invasive evaluation of the tube location and cuff inflation (22). Eventually, double-contrast-enhanced CT scan may even reveal oesophageal injury with mediastinal contrast spreading (23, 26, 27). Despite the valuable information that can be gained through CT, endoscopy remains the gold standard in properly characterizing the tracheal tear and it is mandatory to perform it as soon as possible. Flexible bronchoscopy allows to dynamically evaluate location, length, and depth of the lesion, ruling out involvement of mediastinal soft-tissue or oesophagus and correctly classifying the injury. In mechanical ventilated patients, basic bronchoscopic assessment may fail identifying the lesion, which could be hidden by the cuff or the tube itself; therefore, in such setting, it is recommended to perform a thorough evaluation with cuff deflation and tube manipulation. It is worth underlining that a correct ITI management cannot be planned without performing both CT-scan and bronchoscopy (21, 26).

## Management

ITIs are burdened by significant morbidity and mortality rates (28), which impose an early and efficacious treatment. Nowadays, therapeutic options for management of post-intubation ITIs are the following: conservative, endoscopic, and surgical treatments (27, 28). Currently, definitive indications on best treatment option are still demanded. However, it is broadly recommended to personalize treatment case-by-case, depending on characteristics of the laceration, patient's clinical features, general conditions, and comorbidities, as well as experience of the centre. Main experiences on the management of this disease are reported in Table 1. Traditionally, surgical repair has long been considered the gold standard, praised to be the only procedure preventing mediastinitis or further tracheal scarring stenosis (30, 38). Nevertheless, due to technical difficulties and non-negligible complications rate affecting surgery, there has recently been a shift towards conservative or less-invasive management of ITIs, which has been allowed by development of innovative materials and spread of minimally invasive procedures (27, 30, 39, 40).. Eventually, multidisciplinary assessment is recommended to choose the best treatment option for each patient, invariably depending on his clinical and respiratory conditions.

**Table 1:** Significant studies focusing on management of post-intubation iatrogenic tracheal injuries.

Reference	No of patients	Cause	Site	Lenght	Cardillo grade	Management	Type of treatment	Success rate
Conti et al. (13)	30	Elective intubation 16	Posterior membrane 30	4.5 ± 1.5	NA	Conservative 28	Observation 15	86%
							Intubation 13	
		Emergency intubation 14			—	Endoscopic 0	—	—
					NA	Surgical 2	Posterolateral thoracotomy 2	0%
Cardillo et al. (8)	30	Elective intubation 22	NA	3.2 ± 1.1	I 3	Conservative 29	Glue application 29	100%
					II 24			
					IIIA 2			
		Emergency intubation 8			—	Endoscopic 0	—	—
					IIIB 1	Surgical 1	Posterolateral thoracotomy 1	100%
Schneider et al. (1)	29	Elective intubation 6	NA	4	NA	Conservative 11	Observation 3	100%
		Emergency intubation 10					Intubation 8	
		Tracheostomy 10			—	Endoscopic 0	—	—
		Other 3			NA	Surgical 18	Transtracheal 7	100%
							Posterolateral thoracotomy 11	
Gomez-Caro Andrés et al. (29)	18	Elective intubation 14	Posterior membrane 17	2.83 ± 1.02	NA	Conservative 17	NA	82%
		Emergency intubation 1	Carina 1		—	Endoscopic 0	—	—
		Tracheostomy 3			NA	Surgical 1	Cervicotomy 1	0%
Sippel et al. (30)	13	Elective intubation 4	Posterior membrane 9	4.4 ± 2.9	NA	Conservative 2	Intubation 2	100%
		Emergency intubation 8			—	Endoscopic 0	—	—
		Tracheostomy 1	Membranous-cartilaginous 4		NA	Surgical 11	Lateral thoracotomy 11	73%
Cardillo et al. (16)	62^a^	Elective intubation 51	NA	2.54	I 8	Conservative 55	Glue application 55	100%
		Emergency intubation 11			II 36			
					IIIA 11			
					—	Endoscopic 0	—	—
					IIIB 6	Surgical 7	Posterolateral thoracotomy 5	100%
					IV 1		VATS 1	
							Cervicotomy 1	
Herrmann et al. (31)	64	Elective intubation 19	NA	4	I 2	Conservative 21	Observation 2	100%
		Emergency intubation 17			II 14		Glue application 19	
		Tracheostomy 26			IIIA 5			
		Other 2			—	Endoscopic 0	—	—
					IIIA 23	Surgical 43	Transcervical 29	77%
					IIIB 20		Lateral thoracotomy 14	
Fiorelli et al. (32)	6	Elective intubation 6	NA	3.5	II 3	Conservative 6	Glue application 6	83%
					IIIA 3			
					—	Endoscopic 0	—	—
					—	Surgical 0	—	—
Tazi-Mezalek et al. (33)]	35	Elective intubation 19	Posterior membrane 20	3.74 ± 1.76	NA	Conservative 24	Observation 7	96%
		Tracheostomy 16	Membranous-cartilaginous 14				Intubation 17	
			Anterior wall 1		NA (IIIB 1)	Endoscopic 8	Silicon Y stenting 7	62%
							Oesophageal stenting 1	
					IIIB 3	Surgical 3	NA	0%
Hussein et al. (34)	4	Emergency intubation 3	Posterior membrane 4	5.62	—	Conservative 0	—	—
		Tracheostomy 1			IIIA 4	Endoscopic 4	Nitinol stenting 4	100%
					—	Surgical 0	—	—
Carretta et al. (35)	36	Elective intubation 23	NA	3.5 ± 1	NA	Conservative 16	Observation^b^	94%
		Emergency intubation 7					Tracheostomy^b^	
		Tracheostomy 6			—	Endoscopic 0	—	—
					NA	Surgical 20	Lateral thoracotomy^b^	90%
							Cervicotomy^b^ Transtracheal^b^	
da Silva Costa et al. (36)	2	Emergency intubation 2	NA	5.5	NA	Conservative 0	—	—
					NA	Endoscopic 0	—	—
					NA	Surgical 2	Combined VATS/transtracheal	100%
Welter et al. (37)	17	NA	NA	NA	NA	Conservative 0	—	—
					NA	Endoscopic 0	—	—
					NA	Surgical 18	Endotracheal 18	94%
San Gerardo Hospital^c^	14	Elective intubation 6	Posterior membrane 8	3.14 ± 1.09	II 4	Conservative 4	Intubation 4	100%
		Emergency intubation 4			IIIA 9	Endoscopic 10	Nitinol stenting 10	100%
		Tracheostomy 4	Membranous-cartilaginous 6		IIIB 1	Surgical 0	—	—

NA, not available data.

^a^ 30 patients from previous publication were included.

^b^ Type of treatment is specified, whereas number of patients treated by a specific approach cannot be derived.

^c^ Authors' personal experience.

## Conservative treatment

Conservative approach is widely suggested in asymptomatic patients with small partial-thickness laceration (level I), hemodynamical and respiratory stability, without mediastinal involvement (8, 17). However, indications to conservative management are now spreading to larger (up to 9 cm) or even deeper (up to level IIIA) tears (30, 41). Conservative options consist of observation, intubation, tracheostomy, fibrin glue application. Whatever the chosen conservative technique, strictly follow-up of patients is of paramount importance to early detect any clinical worsening.

• Observation is based on rest, antitussive drugs, and broad-spectrum antibiotics. This management may be adopted in case of small (< 2 cm) level I tears in asymptomatic or pauci-symptomatic patients (40).
• Intubation allows to overcome the injured segment, by placing the cuff distally in healthy tissue, ensuring ventilation support (22, 27). This management may be adopted in case of level I-IIIa tears in patients needing for ventilation. Anyway, ventilator setting should provide protective ventilation, by minimizing airway pressures. If the length of the lesion does not allow to place the cuff distally, after considering a double-lumen tube, and the respiratory failure is not otherwise manageable, ECMO support is worth to be accounted (42).
• Tracheostomy (40, 43) is considered a fallback option, due to significant side effects, but may be indicated in long level I–II tears, since it decreases endotracheal pressure ensuring progressive healing of the injury.
• Glue application is an innovative procedure proposed by Cardillo and colleagues (8); it consists of instillation of fibrin sealant to directly cover the tear through flexible bronchoscopy. It is generally appliable in level I–IIIA tears. Recently, the same authors (16) have presented an updated series of 55 patients treated by glue application, showing 100% success rate, when the procedure is performed in experienced centres on fit patients.

## Endoscopic treatment

Several cases of patients with endoscopically managed ITIs have been reported in literature with encouraging results (40, 41). In the recent past, this option was reserved to poor surgical candidates (12), deemed unfit for surgery, due to comorbidities. The reported satisfactory results have prompted some physicians to spread the indications for this technique (2, 27, 40). Nowadays, endoscopic treatment may be suggested for treating level IIIA or even selected IIIB lesions instead of surgical approach, in patients with worsening clinical conditions, such as expanding pneumomediastinum/subcutaneous emphysema, high risk for mediastinitis, if without signs of actual mediastinitis, or prolonged mechanical ventilation without short-term perspective of weaning (40, 41, 43–45). The technique consists of rigid bronchoscopy and temporary placement of covered metallic or silicone stent over the laceration, allowing for granulation tissue to close the defect. It is suggested to keep the stent in position from 4 to 8 weeks, then it can be removed (46). This procedure is inherited by lung transplantation field, where it is applied in case of post-transplant tracheobronchial dehiscence (44). Complications reported in literature include stent migration, tracheal stenosis, mucus plugging and local infections (44, 47, 48). If benefits overcome these risks, stenting could be a valid surrogate of surgery, allowing for bridge treatment and delaying surgical approach after improving general conditions of the patient, or even for definitive repair, ensuring lower morbi-mortality rates especially in high-risk surgical candidates (34, 49, 50). The placement of nitinol-coated self-expandable metallic stents (n-SEMS) seems to be particularly interesting (33, 41, 43, 45) since it could apparently fit better in the airway than silicone ones, decreasing the risk of migration, while preserving the tracheal segment from air leakage.

## Authors' personal experience

**Figure 1:**
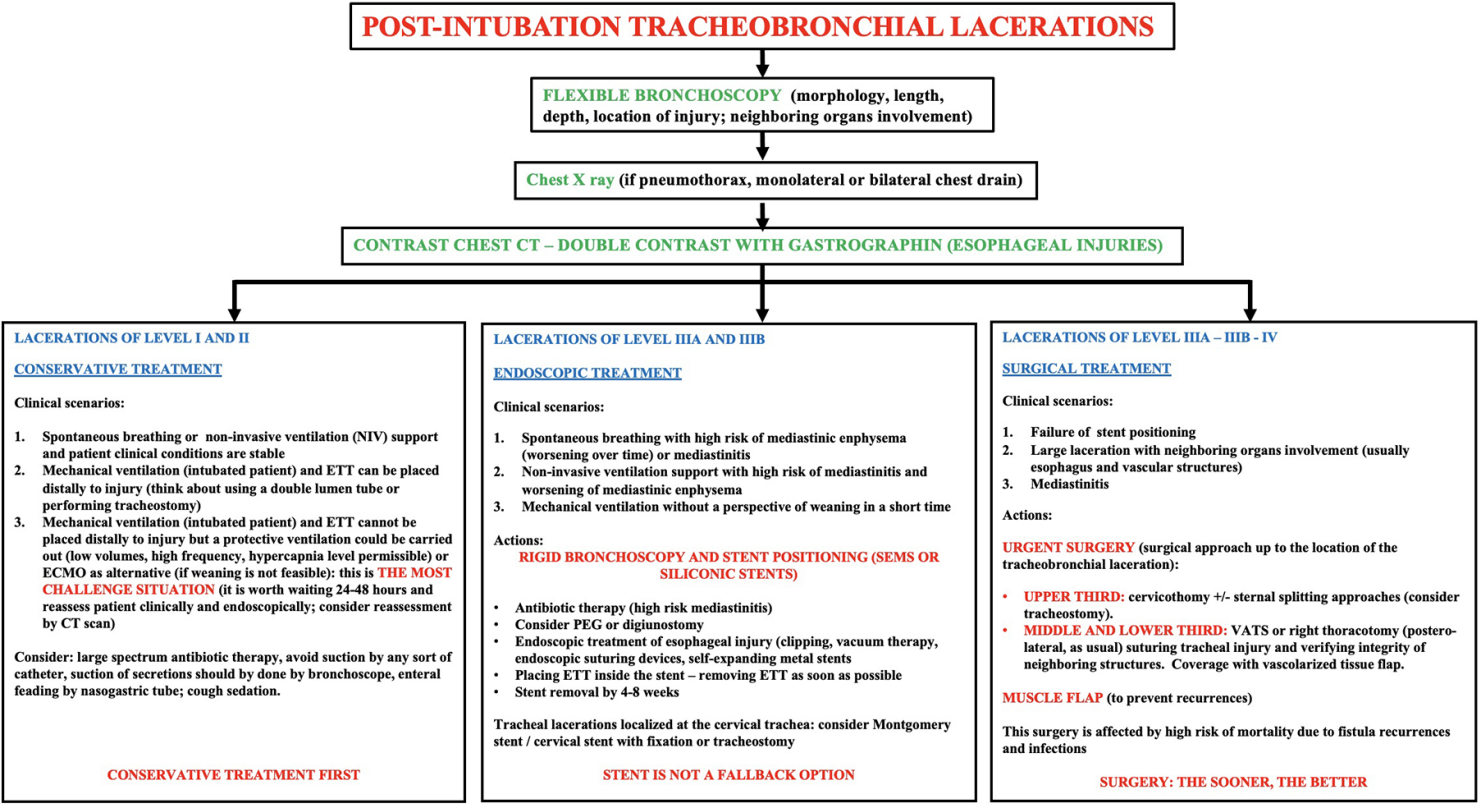
Diagnostic-therapeutic algorithm proposal for management of post-intubation tracheobronchial injuries.

In the last three years, 14 patients with post-intubation ITIs were referred to the Department of Thoracic Surgery of our tertiary centre (San Gerardo Hospital, Monza, Italy). The injury was due to endotracheal tube mispositioning in 10 patients and emergency tracheotomy in 4 patients. It was along the tracheal posterior membrane in 8 cases (57%) and at the tracheal membrane-cartilaginous junction in the remaining patients. 4 lacerations were classified as level II, 9 as IIIA, 1 as IIIB. Upon multidisciplinary discussion, we have successfully treated all the patients, through conservative or endoscopic treatment, depending on patients' clinical and respiratory conditions, according to the in-hospital protocol reported in Figure 1: 4 patients (level II) were conservatively treated, 10 patients (9 IIIA and 1 IIIB) were endoscopically managed. The conservative treatment consisted of endotracheal tube proper positioning and observation. On the other hand, the endoscopic treatment consisted of n-SEMS placement through rigid bronchoscopy, within 72 h from detection of ITIs; 30-day morbidity and mortality rates were null, and the stent was removed 4–6 weeks later without complications. All the injuries were completely healed at 1-month, without any relapse at 6-month follow-up. We were prompted to endoscopically handle patients with level III injuries, instead of adopting a conservative strategy, because of their respiratory conditions: all the patients were still ventilatory-dependent, due to primary lung failure, without a perspective of weaning in short time. In such setting, we strongly believe that a conservative treatment could be hardly feasible.

## Surgical treatment

Surgery is recommended for highly symptomatic patients with large level IIIA, especially in case of ineffective mechanical ventilation, or level IIIB lacerations, mainly when involving vascular structures or esophagus, as well as for level IV tears, or any lesion occurring with mediastinitis (22, 35, 38). Most authors agree that fit patients with rapidly worsening clinical conditions, despite previous conservative or endoscopic treatment, should undergo surgery, preferably within 48–72 h from the original event, to mitigate morbidity and mortality rates (2, 35, 50). Different surgical approaches are described in literature (2, 12): open, video-assisted thoracoscopy surgery (VATS), and endotracheal. The decision to perform one rather than others approach relies on the site and extent of injury, the emergency or elective setting, the experience of the center. Open techniques consist of posterolateral right thoracotomy (28), which was traditionally the approach of choice for emergency procedures and for middle or lower thirds tracheal injuries, and cervicotomy, as introduced by Angelillo-Mackinlay (51) in case of upper third lesions, possibly associated with sternal split if middle third is involved. VATS techniques include right thoracoscopy, as well as video-assisted transcervical-transtracheal approach, which was proposed by da Silva Costa and colleagues (36) introducing an endoscopic needle holder and a 0-degree camera though the tracheal incision. Either in open or VATS approach, continuous running or interrupted sutures are used, based on surgeons' choice. To prevent recurrences or fistulas, mainly in case of mediastinal inflammation or infection, pedicled muscle flaps are placed over the suture line (50). Another promising technique is the endotracheal repair, firstly described in 2011 by Welter and colleagues (37). It is performed using an endoscopic needle holder through rigid tracheoscopy, leading to a totally intraluminal repair, with lower surgical trauma and postoperative pain (37).

## Conclusions

Post-intubation ITIs are rare complications of intubation or tracheostomy, nevertheless they are clinically significant due to their high morbidity and mortality rates. Keeping high clinical suspicion is of utmost importance, and patients with suggestive symptoms should early undergo thorough diagnostic workup, through radiologic and endoscopic assessment to detect and characterize the suspected injury. The management of post-intubation ITIs is still a matter of debate and definitive guidelines are still lacking. Procedural and instrumental innovation, as well as medical development, have likely revolutionized traditional management of post-intubation ITIs, broadening the use of conservative treatment and introducing the opportunity of endoscopic approach, with interesting success and reasonable complication rates. In such setting, endoscopic stenting may be a viable alternative to surgery and no more a fallback option, limiting surgical management to advanced stages or in case of failure of other treatments. On the other hand, surgery has become less and less invasive, leading to lower morbidity and mortality rates than in the past. Patients' general clinical and respiratory conditions must be considered in the management pathway. Anyway, multidisciplinary evaluation and personalized treatment of each patient at experienced centres are strongly recommended.

## Data Availability

The raw data supporting the conclusions of this article will be made available by the authors, without undue reservation.
